# Including older rural adults in research: Practical guidance for addressing the NIH Inclusion Across the Lifespan policy

**DOI:** 10.1017/cts.2020.12

**Published:** 2020-02-13

**Authors:** Raina Croff, L. Kris Gowen, Allison Lindauer, Sabrina Shofner, Kim Brown, Elizabeth Eckstrom

**Affiliations:** 1 NIA-Layton Aging and Alzheimer’s Disease Research Center, Oregon Health & Science University, Portland, OR, USA; 2 Oregon Clinical and Translational Research Institute, Oregon Health & Science University, Portland, OR, USA; 3 Division of General Internal Medicine & Geriatrics, Oregon Clinical & Translational Research Institute, Oregon Health & Science University, Portland, OR, USA

**Keywords:** Research participation, underrepresented populations, perceptions of research, decision-making, rural health, older adults, NIH policy

## Abstract

**Introduction:**

The NIH Inclusion Across the Lifespan policy has implications for increasing older adult (OA) participation in research. This study aimed to understand influential factors and facilitators to rural OA research participation.

**Methods:**

Thirty-seven rural adults aged ≥66 years participated in focus groups in community centers in four Oregon “non-metro” counties. Transcribed discussions were coded using open-axial coding by an interdisciplinary analytical team.

**Results:**

Ages were 66–96 (mean 82.2) years. Majority were women (64%) and white (86%). Primary, interrelated discussion themes were Motivation and Facilitators, Perceptions of Research, and Barriers to Research Participation. Participants were motivated to engage in research because they believed research had implications for improved longevity and quality of life and potentially benefited future generations. Motivational factors influencing participation included self-benefit and improving others’ lives, opportunities to socialize and learn about current research, research transparency (funding, time commitment, and requirements), and financial compensation. Perceptions influencing trustworthiness in research included funding source (industry/non-industry) and familiarity with the research institution. Barriers to research participation included transportation and concern about privacy and confidentiality. Suggestions for making research participation easier included researchers coming to rural communities and meeting participants in places where OAs gather and providing transportation and hotel accommodations.

**Conclusion:**

Lessons learned offer practical guidance for research teams as they address the new NIH Inclusion Across the Lifespan policy. Including OAs in research in ways that motivate and facilitate participation will be critical for a robust representation across the lifespan and in tailoring treatments to the specific needs of this population.

## Introduction

In January 2019, the National Institutes of Health (NIH) adopted the Inclusion Across the Lifespan Policy mandating that “individuals of all ages, including children and older adults (OA), must be included in all human subjects research (45 CFR 46). Applications/proposals must include a description of plans for including individuals across the lifespan” (https://grants.nih.gov/policy/inclusion/lifespan.htm) [1]. This policy will impact all research teams that study widespread chronic diseases which occur in older and rural adults (e.g., heart disease, cancer, diabetes), and all research team members will need to address challenges to inclusion of OAs in their study design, recruitment, and retention.

Many diseases are common in older people and in people living in rural areas, yet little research has been done in these populations. A recent study of phase 3 clinical trials using data from www.clinicaltrials.gov showed that the mean age for studies on congestive heart failure (*N* = 35), coronary atherosclerosis (*N* = 81), heart attack (*N* = 63), stroke (*N* = 67), and lung cancer (*N* = 55) was 61, 59, 58, 53, and 52, respectively [2]. Yet, as an example, at least 35% of coronary atherosclerosis is diagnosed in people over 75 years. Any primary care clinician will assert that they regularly see patients with these diseases in their 80s, 90s, and even 100s and recognize they have little research to guide care in these age groups. Twenty-one percent of people over 65 years have diabetes, yet of 440 diabetes trials, 66% excluded OAs with an arbitrary age cut-off and 76% excluded those with comorbidities [3]. For ischemic heart disease, the estimated proportion of subjects aged 75 and older was 12.3%, yet we know that about 39% of ischemic heart disease occurs in people over 75 years [4]. Researchers who focus on diseases of OAs, such as Alzheimer’s disease and related dementias, may have the most experience in inclusion of OAs in research. Despite this, and while 72% of people with Alzheimer’s disease are over 80, the mean age of participants in dementia trials was 74. Only 8% of clinical trial participants were over 85 years [5]. In addition, many diseases are distinctly different in OAs, due to poor renal clearance, lower muscle mass, effects of multiple comorbidities, and phenotypic variation (e.g., atherosclerotic heart disease) yet have not been studied in these age groups [6].

Similarly, rural adults have been underrepresented in research. A 2014 telephone survey in South Carolina identified that rural residents were more likely than urban residents to perceive limited access to clinical trial sites, as well as lack of awareness of available trials [7]. A 2015 focus group study done by the same group in South Carolina showed that rural residents believed that clinical trials involved deception more often than urban residents and expressed that their participation would depend on whether their doctor recommended it or whether the trial would benefit a family member’s health [8]. A 2018 Arkansas study showed that 45% of adults would be willing to participate in health research and rural respondents did not differ from urban respondents on this, though only 8.5% of rural respondents reported that they had participated in research [9]. Nation-wide data on the actual inclusion of rural OAs in research are lacking.

The Clinical & Translational Science Award Program (CTSA), which has recently incorporated a new Integrating Special Populations Core, is uniquely positioned to provide expertise to research teams in including older and rural adults in research. As part of the Oregon Clinical & Translational Research Institute (OCTRI, Oregon’s CTSA) and in collaboration with the Oregon Health & Science University (OHSU) Layton Aging and Alzheimer Disease Research Center (NIA-funded), we conducted focus groups with older, rural Oregonians to determine: (1) barriers that limit inclusion of rural OAs in research and (2) strategies that potential research subjects feel would help enhance the number of rural OAs who are included in research.

## Methods

This study was approved by the OHSU Institutional Review Board prior to participant enrollment. Participants were not required to sign informed consent but received an information sheet prior to participation and were given the ability to opt out after reading it.

## Subject Recruitment

OCTRI’s Community Research Hub assisted in recruiting rural adults aged 66 and older to participate in focus groups via rural community centers around the state. OCTRI Community Research Hub employees approached site directors at community centers in four rural Oregon counties (designated as 10 miles or more from a population center of 40,000 people or more) [10]. Site directors hung flyers at the community center encouraging participation in the focus groups and kept a sign-up list for each focus group. Focus groups were held after another desirable activity (such as lunch) to encourage attendance. No site directors refused to participate. OA participants received a $25 gift card to a local business.

## Study Design

Focus groups were held in four different Oregon “non-metro” counties; three counties were rated 6 and one 4 according to the USDA’s Rural Urban Continuum Codes (ratings range 1–9, where a higher number represents smaller communities) (https://www.ers.usda.gov/data-products/rural-urban-continuum-codes.aspx) [11].

This qualitative study utilized 60-minute semi-structured focus groups of rural OAs (*n* = 37) to develop themes around barriers and facilitators for the inclusion of older rural adults in research. Participants were asked about their experience with research, biases toward or against participating in research, and suggestions they had to make it easier to participate in research. Focus group facilitators were specifically trained to ensure that all participants expressed their opinion, so that results would not be representative of only a few subjects. Focus groups were audiotaped and transcribed.

## Analysis

Authors consisted of a qualitative methodologist with academic training in developmental psychology with expertise in qualitative methodologies (LKG), a geriatrician and aging researcher (EE), a medical anthropologist with training in qualitative methodology (RC), a nurse scientist with dementia expertise and a qualitative background (AL), and a qualitative analyst in training (SS). Together, the authors comprised an interprofessional team whose varied experiences provided opportunities for diverse interpretations of the data, supporting efforts to mitigate potential coder bias.

One focus group transcript was read by all authors to identify codes and general concepts to apply to remaining data; these codes were revised and refined at a series of collaborative meetings among all authors. This open-axial coding process identified emergent themes [12] where authors engaged in conversations about their independently generated codes before collaboratively finalizing the coding framework (including emergent and a priori codes). Primary codes emerging from this process included how participants felt about medical research, as well as motivations and barriers for participating.

Then, three pairs of authors each coded one to two transcripts in the qualitative analytical program Dedoose [13]. Coders were instructed to apply the coding framework, examine uncoded text, and look for negative cases. For each transcript, a primary coder was assigned and a secondary coder reviewed and noted discrepancies in code applications; this process served as both a validity and quality check [14]. The first and second coders then met to resolve discrepancies, which were coded as “Questions” in Dedoose in order to document their occurrences. Finally, the entire team came together in a series of meetings to refine themes and identify the best examples from the data to represent themes.

## Results

**Fig. 1.**
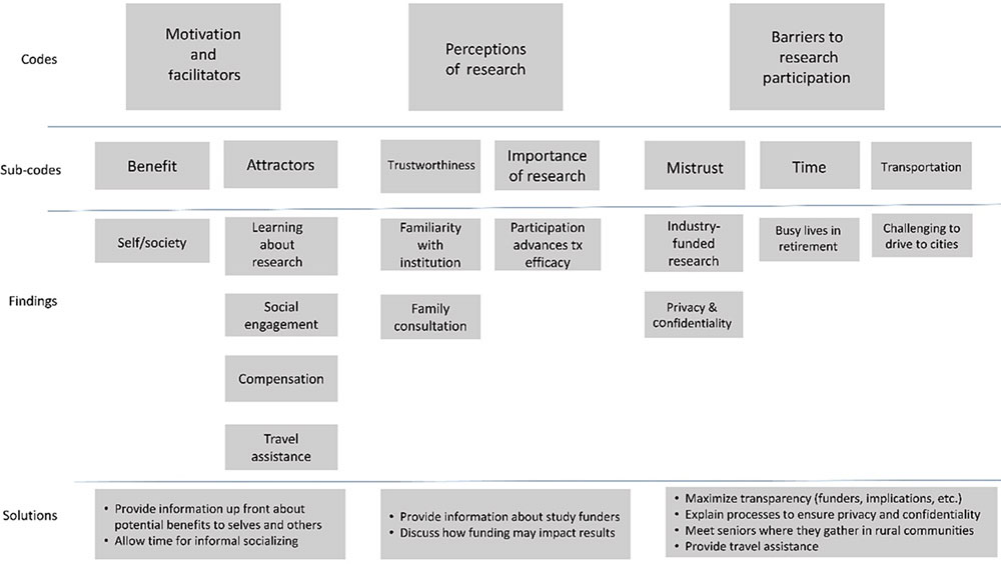
Hierarchical code scheme, findings, and solutions.

Participants were aged 66 (*n* = 1), 70–74 (*n* = 3), 75–79 (*n* = 11), 80–84 (*n* = 9), 85–89 (*n* = 4), and 90+ (*n* = 9). Mean age was 82.2 years. Of the 37 participants, 86% reported race as white and 42% reported having a bachelor’s degree or higher (compared to 20% reporting a bachelor’s degree across rural Oregon, though this statistic was not specific to OAs [15]; Table 1). Recurrent themes were Motivation and Facilitators, Perceptions of Research, and Barriers to Research. Representative quotes from each of the four OA focus groups illustrate themes. Table 2 and Fig. 1 outline themes, definitions, and summary of findings.

**Table 1.** Focus group demographics

Total (*n* = 37)	Male (*n* = 13)	Female (*n* = 24)
Age, mean (range)	80.5 (66–92)	83.14 (70.5–96)
Gender (%)	36	64
Education		
% Bachelors	30	20
% Graduate and higher	23	16
Insurance (% yes)	100	100
White (%)	84	87
Black/African American (%)	0	0
Asian (%)	7	8
Native Hawaiian/Other Pacific Islander (%)	0	0
Hispanic/Latino (%)	0	0
Native American/Alaskan Native (%)	0	0
>1 Race (%)	0	8

**Table 2.** Discussion themes and summary of findings

Theme	Sub-theme	Definition	Summary of findings	Solutions
Motivation and facilitators	Self-interest/individual experience	Research helps participant directly. Experience with the condition or friend/family experience with the condition	Benefitting self Research improves longevity and quality of life	Provide enough information up front for potential subjects to understand and recognize the potential benefits to themselves and others
	Helping others/society	Hopefulness/anticipation about participation benefitting the next generation, helping to find a cure or treatment, or deeper understanding of a condition or pathology	Benefitting future generations Helping science	
	Participant attractors	Factors leading to research participation and sustained engagement; favorable aspects of the research experience, and reasons for involvement	Learning about research Research transparency Financial compensation Social engagement	
	Money	How researchers or research institutions made participating in research easier, more convenient, or more desirable because of providing compensation	Cash compensation Travel reimbursement Hotel accommodations Gift cards and fuel cards	Ensure compensation for travel and other expenses Provide a small honorarium
Perceptions of research	Lack of trust in research	Factors influencing lack of trust in research, the research team and/or host facility	Pharmaceutical/industry funding Lack of familiarity with researcher/institution Beliefs about unethical practices	Provide enough information for potential participants to fully understand who is supporting the study and how study funding might impact results
	Trust in research	Factors influencing trust in research, the research team and/or host facility	Familiarity with the institution Vetting research through family in healthcare field Belief in God’s hand in medical research	
	Research importance	Factors regarding research importance that influence participation	Broadens pool of people testing treatments for wider efficacy	
Barriers to research participation	Barriers – general	Factors, logistics, situations that made participating in research more difficult, including concerns about participation	Mistrust in industry-funded research Privacy and confidentiality Time commitment/busy lives	Maximize transparency and discuss how study funding might impact results. Explain the processes in place to ensure privacy and confidentiality
	Transportation	Getting to and from the research location. Includes navigation – how to find the location	Challenging to travel to cities to research locations Desired study-provided assistance	Come to rural locations, meet seniors where they gather Provide town car/bus

### Theme 1: Motivation and Facilitators

Discussion coded under this theme spoke to what motivated participants to engage in research, including factors that led them to seek out research participation, accept invitations to participate, and stay engaged once they began. Facilitators were factors that made participation easier.

Overall, older rural adults felt medical research has helped improve their lives, has positive implications for society, and has great potential to benefit future generations. Older rural adults felt strongly that medical research has helped them live longer and better, as one participant explained, “I think most of us here have artificial parts. And all of us are the result of research” (OA1). The impact of research on the quality of life was also important: “Yeah, I’m concerned about the quality of my life and if there was something that I can be doing which would make that quality improve, I would be so motivated to be a part of it” (OA1). OAs were motivated to participate in research to help themselves, “It’s important that we have medical research and that we find some results and it will hopefully make a difference in my life” (OA2). Additionally, participants found value in learning about current research through participation: “Because I want more information and so I want to help” (OA2). Older rural adults saw their individual research participation as contributing to the collective good of society: “…we’ve got to think of the overall results…what we’re going to do for future generations” (OA2) and were motivated by the potential long-term impact, “Research that’s going on right now could possibly not affect me, but could be helpful to my children or children’s children…” (OA2).

Research for the sake of research itself was also important, recognizing that research can lead to medical cures and living longer, “I started having a heart problem at 36 and they didn’t think I’d live to be 50. Now I’m 75 so research is wonderful” (OA4). Another participant simply stated, “Helping science. That motivates” (OA2).

Alongside believing that research helps themselves and others, financial compensation, not surprisingly, motivated older rural adult research participation: “Well, if you have time and travel expenses, you know, or having to be away from home, certainly money comes into it” (OA1). Providing stipends, reimbursement for travel, and hotel accommodations were all ways participants suggested to mitigate transportation barriers that might otherwise impede participation. Additionally, gift cards and fuel cards were mentioned as positive ways to provide financial support.

Social drivers to participation also factored into older rural adults’ decision-making processes to join research studies, seeing participation as a way to meet others and share the experience with their friends. One participant noted that longitudinal studies with ongoing recruitment may be less restrictive and could allow current participants to facilitate the recruitment process by spreading the word about their positive research experience: “If it was an ongoing study and they could join at any time, that would be ideal because then they would have their friends to tell them–‘Hey, we’re having fun. You might learn something. Why don’t you come in and join us?’” (OA4).

Participants were advised using multi-modal advertising strategies to spread information about the study; flyers, advertisements in local papers, and Internet-based information were all perceived as helpful.

Research transparency was important for older rural adults and facilitated their decision-making regarding research participation. They wanted to know what would be expected of them and who was behind the research before they decided to commit to a study. As one participant explained, “Just not knowing sometimes. You know, what’s going to happen or anything else” (OA4). Rather, participants desired a clear explanation about what committing to the research would mean for them. Participants also recognized their own role in finding out more about the researchers and the research: “You’d have to study the researchers. You have to have a background for that – no matter what you’re investigating” (OA4). Another participant remarked on the value of transparency to increase OA participation: “I think just a good explanation of the importance would encourage a lot of people to participate” (OA4).

### Theme 2: Perceptions of Research

This theme captured any beliefs, perceptions, or ideas formed about research and institutions as the result of experiences as a research participant. Perceptions and beliefs about research shaped participants’ attitudes about research and participation and thus influenced decision-making processes about joining research. Emergent sub-themes were trustworthiness and importance of research. Trustworthiness was addressed in terms of factors that contributed to a lack of trust and factors that curated trust.

One group had a lengthy conversation about distrust in research because of what some believed was the role that the pharmacology industry played in influencing research, “[Pharmaceutical companies] are only interested in making money and like cancer, chemotherapy, right? It never has worked, but they keep doing it. Why; because they make a lot of money off it” (OA2). Participants in this group wanted wider access to a variety of research-based information not tied to large corporate funding. Similarly, another group had individuals who believed profit, not people, was the primary concern of some research. In discussing doctors filling prescriptions for opiates in medication-assisted treatment for drug addiction, one participant summed up the process as: “Money – that’s what drives the machine” (OA3).

The group that discussed the pharmacology industry and corporate influences on research also believed these entities caused medical training to be biased toward medications, which in turn was part of why research was so heavily biased toward pharmaceutical studies. Rather, participants in this group wanted to see doctors provided with a wider training in health beyond medications. As one participant explained, “…medicines have so many side effects that you’re almost scared to take them, but then you’re afraid not to…” (OA2). Further, participants in this group desired to have traditional medical doctors and naturopaths work more closely together, “We do need doctors, M.D.s as well as we need naturopaths, but…I’d like to see them, the two of them, meet” (OA2). Finally, beliefs about the existence of unethical practices in research contributed to lack of trust in research.

Trust in research was fostered by institutional recognition, as one participant explained, “You have more faith in those institutions that are doing research that you recognize or have heard of” (OA3). One participant mentioned vetting research through trusted family members working in the healthcare field.

Research was seen as important because it could lend insight into genetic factors that influence treatment effectiveness. Participants understood that treatments worked differently for different people, and so participating in research was important because it widened the pool of people testing treatment efficacy, “…if everybody here had the same thing and you all have the same treatment, it would work differently with each person and that’s where the research learns a lot of things…” (OA3). When asked if they had much faith or trust in medical research, one participant responded with others agreeing, “We depend on it” (OA3).

Just as positive perceptions of research formed the basis of many motivators, negative perceptions about research were tightly tied to barriers to participation and were often at the root of conceptual reasons why some older rural adults would not participate in research.

### Theme 3: Barriers to Research Participation

This theme captured any factors, logistics, and situations that made participating in research more difficult, including concerns about participation.

Similar to findings about perceptions in research, older rural adults saw mistrust of the research community as a barrier to research participation. Specifically, two groups voiced animosity toward “big pharma;” a third group did not name “big pharma” specifically but did hold similar beliefs that money, not people, drove research. As one participant said about pharmaceutical companies, “I wouldn’t want to help them make more money” (OA4). Participants were leery of pharmaceutical companies’ motivations. They were worried that the pharmaceutical companies focused only on profit and would go to extremes to make the profit: “It’s difficult to know the difference between authentic studies and those that are developed for advertising” (OA1).

Privacy and confidentiality were also concerns, regarding who was using the information collected in research and how. Participants spoke of discomfort in “being followed around” like a “lab rat” (OA1).

Although most participants were retired, they led busy lives and had to make choices about how to spend their time. Participating in research was seen as time-consuming for some: “There are limitations to your life and how you want to serve” (OA3). Participants did not want to “waste my time” (OA2) on paperwork or be forced to conform to the needs of study demands and schedules.

Transportation was a common barrier. Participants wanted options to driving to study sites in large cities: “I think the major challenge would be getting to and from where they needed you to be…it takes forever to get up there [to the study site]” (OA1). Participants spoke of options to reduce transportation barriers. Participants’ suggestions included study staff driving to meet participants at senior centers, having the study pay for a town car or bus to transport participants, and, for those traveling greater distance to participate, providing hotel accommodations. Of interest, none spoke of using tele-commuting technology (even a phone) to bridge the geographic gaps.

## Discussion

Adults over 65 and from rural America are one of the least represented groups in research. This study qualitatively explored perceptions of this underrepresented group and offers insight into how research teams can improve their inclusion in research trials. Three interrelated themes were reported: Motivation and Facilitators, Perceptions of Research, and Barriers to Research. Themes overlapped, attesting to the intersectionality of factors influencing older rural adults’ decisions to participate in research.

Older rural adults perceive multiple barriers to research, including mistrust in research, a need to protect privacy and confidentiality, time commitment, and transportation issues. Participants offered several relatively easy solutions to these barriers including ways to mitigate transportation barriers. Others noted more complicated barriers that deal with changing deeply-held negative perceptions about research, like dispelling suspicions about hidden agendas related to the pharmacologic industry. Less complicated than changing perceptions but still not an easy fix, will be efforts put forth by lesser-known research institutions to gain the trust of older rural adults.

What have we learned from this study that adds to the literature on inclusion of older and rural adults in research? Individuals across the age spectrum, including advanced ages, who pragmatically recognize that participating in research may no longer hold value for their health, feel emphatically that they should participate in research to help future generations. This group recognizes that including people like themselves in research can broaden and deepen medical knowledge. They appreciate the social aspect of being part of a research trial and appreciate the purpose that participating in research provides. They want transparency in research and want the research to be free of bias such as that which funding by industry might imply. They want research teams to make participation in research easy, such as coming to rural communities or providing transportation and hotel costs. These findings can help shape recruitment materials and research approaches to make studies more appealing to OAs.

## Limitations

Limitations in this study of rural OAs include lack of identification of individual participants, which hopefully enhanced honesty and forthrightness but did not allow us to know exactly how many participants shared the perceptions that emerged. In addition, Oregon is not racially and ethnically diverse, and this study reflected that with 86% of our study participants reporting being of Caucasian background. The percentage of participants reporting a bachelor’s degree or higher (42% vs. statewide statistics of only 20%) may have introduced bias in the responses (as people with a higher education level may be more willing to participate in research). Our recruitment method itself (voluntary participation at local community centers) may have introduced bias because those less positive about research in general would not have chosen to take part in the focus groups. We only studied rural OAs, and few research exists in urban populations, so we cannot guide researchers on how they may need to approach rural OAs differently from those in urban areas. We suspect urban themes would be similar to ones we described, with possibly less emphasis on the challenges of transportation.

## Conclusions

This qualitative study of rural adults over age 65 helps provide realistic guidance to research teams as they strive to implement the new NIH Inclusion Across the Lifespan policy. OAs are important for research to tailor treatments, services, recommendations, and devices to their specific needs. Researchers need to consider the particular research participation motivators and barriers important to rural OAs. They need to maximize transparency in research projects, such as providing potential subjects with full information about study funding. This underrepresented group of rural OAs does want to be included; it is now the job of the CTSAs and other research leaders to help ensure that all research teams have the expertise and resources to fully implement this important new policy.
